# Fallback Variable History NNLMs: Efficient NNLMs by precomputation and stochastic training

**DOI:** 10.1371/journal.pone.0200884

**Published:** 2018-07-26

**Authors:** Francisco J. Zamora-Martínez, Salvador España-Boquera, Maria Jose Castro-Bleda, Adrian Palacios-Corella

**Affiliations:** 1 R&D Department, Veridas S.L., Pol. Ind. Talluntxe II, Tajonar 31192, Spain; 2 Universitat Politècnica de València, Valencia, Spain; Universita degli Studi di Siena, ITALY

## Abstract

This paper presents a new method to reduce the computational cost when using Neural Networks as Language Models, during recognition, in some particular scenarios. It is based on a Neural Network that considers input contexts of different length in order to ease the use of a fallback mechanism together with the precomputation of softmax normalization constants for these inputs. The proposed approach is empirically validated, showing their capability to emulate lower order *N*-grams with a single Neural Network. A machine translation task shows that the proposed model constitutes a good solution to the normalization cost of the output softmax layer of Neural Networks, for some practical cases, without a significant impact in performance while improving the system speed.

## 1 Introduction

Neural Network Language Models (NNLMs) have drawn the attention of the Natural Language Processing community due to their ability to learn continuous word representations, which allows for a better generalization than that of count-based *N*-grams by using either feed-forward or recurrent Neural Networks (NNs) [1–5]. Speech recognition, handwritten recognition and machine translation are good examples of tasks where NNLMs have shown to improve performance.

### 1.1 Neural networks language models

The aim of a Language Model (LM) is to estimate the probability of a word *w*_*i*_ given the previous ones, *p*(*w*_*i*_|*w*_1_ …*w*_*i*−1_). An NNLM is a language model based on NNs, exploiting their ability to learn continuous word representations [1, 2, 6–8]. Feed-forward neural networks are used for language modeling based on the *N*-gram approximation since the past history of word *w*_*i*_, defined as *h*_*i*_, is limited to the *N* − 1 previous words: *p*(*w*_*i*_|*h*_*i*_) = *p*(*w*_*i*_|*w*_*i*−*N*+1_ …*w*_*i*−1_). In these NNLMs, the input is composed of the sequence *w*_*i*−*N*+1_, …, *w*_*i*−1_. For a given vocabulary Ω, words are represented by a one-hot or 1-of-|Ω| vector, leading to a huge NN for large vocabulary tasks. To overcome this problem, a word embedding can be learned by means of a projection layer, mapping each word into a lower-dimensional real-valued space and sharing the weight matrix [1, 2] (note that the projection layer can be later replaced by a table storing the learned distributed encoding of each word). There exist, nevertheless, other alternatives to estimate the local embedding [6], and the chosen procedure is irrelevant for the approach described in this work.

After this input and the optional projection layer, there can be one or more hidden layers with non-linear activation functions. The last hidden layer is connected to the output layer, usually based on the softmax function to represent the *N*-gram LM probability distribution:

$$\begin{array}{c} p_{j}=P\left(w_{i}=j|h_{i}\right)=\frac{exp(o_{j})}{\sum_{k=1}^{\left|\Omega \right|}exp\left(o_{k}\right)} \end{array}$$ (1)

where *o*_*k*_ is the output of output neuron *k* before applying the softmax normalization.

The computational complexity of this NNLM is dominated by the size of this output layer, which needs one neuron for each vocabulary word. This becomes a bottleneck, even if only a few outputs are required, since the computation of the normalization factor requires the sum of all output neurons.

### 1.2 Previous work

Different approaches have been proposed to overcome the computational cost of computing the normalization term during training. Among them, we can mention importance sampling of the partition function [9] and the Noise-Contrastive Estimation [10]. On the other side, the shortlist approach [2, 11] and hierarchical NNLMs (also called structured output NNLMs) [12, 13] reduce the computational cost of evaluating the models by limiting the size of the softmax layer either by shortening the output vocabulary, or by using some kind of tree structure to hierarchically distribute words into classes, respectively.

While some other approaches elude computing the softmax denominator by training the network with a regularization term to reduce its variance [14, 15], ignoring these normalization constants still degrades the model [16]. We can conclude that these computational issues are not completely solved yet. Indeed, none of the aforementioned solutions has been adopted as the best or as standard by the community.

Relating the reduction of the computational cost during evaluation, we can distinguish between two different scenarios: on the one side, we may need to compute the whole set of outputs and, on the other side, only a few outputs are often required. In the first case, the computation of the partition function is not the bottleneck and approaches such as the hierarchical output may be counterproductive: only approaches such as the differentiated softmax [17], when the overall number of arithmetic operations is reduced, can make a difference.

Let us also observe that, if we are only interested in determining which are the most probable outputs of the network, without requiring their actual normalized values, we can apply several techniques to speed up this process since it is essentially the search of a maximum inner product [18].

The computation of the softmax denominator term is more problematic in the second scenario, where only a little subset of the overall output must be computed in spite of the fact that the rest of the outputs should also be computed, in principle, only to obtain the normalization term.

The description of some practical cases where computing a limited subset of outputs makes sense illustrates the relevance of this scenario: in a speech or in a handwriting recognition task based on HMMs, the system is guided by an LM. This LM can be based on sub-word units (e.g. character-based LMs) or, more usually, word-based. In these word-based systems, the lexicon decoder (e.g. a tree lexicon) proposes a set of word hypotheses whose number is usually much lower than the vocabulary size (e.g. only a few part of the vocabulary is activated each time), so that the LM look-ups are only required for this limited subset. Moreover, in order to properly apply dynamic programming, and due to the fact that hypotheses from different alignments are taken into account, LM scores should be normalized, which makes the use of the softmax normalization unavoidable (for NNLMs using this output layer).

Besides HMMs, more recent recognition systems based in deep learning techniques usually employ the Connectionist Temporal Classification (CTC) loss function [19]. In spite of the fact that CTC based systems are able to emit characters or other units in the absence of an LM, it is possible to include them in several ways either by constraining the paths and using some kind of beam search (instead of greedy decoding) or even by means of re-ranking over the best hypotheses [20]. Similarly, the use of an LM can also be adapted to the encoder-decoder approach usually employed for automatic translation [21].

### 1.3 Proposed approach

Our work, aiming at reducing the computational cost during evaluation, is based on precomputing some values beforehand. The idea of precomputing values for this purpose is not new: it has been applied to the first hidden layer of NNLMs [15] and to the output layer as well [7, 22, 23]. In [22], a fast locality-sensitive hashing technique is used, together with a sublinear nearest neighbor search, to determine a set of outputs with the highest probabilities. The normalization factor is then computed by discarding the values not present in this set. [23] also proposes the use of approximate nearest neighbor search but makes use of kernel feature maps.

In [7], softmax normalization constants are directly stored for some inputs (e.g. the most probable ones from those appearing in the training set). When an input pattern is not found, we can either compute the denominator on the fly or, much faster, we can apply a fallback mechanism consisting in using a lower order *N*-gram model. This last approach reminds us the backing off mechanism commonly used in count-based *N*-gram LMs [24].

A more extreme precomputation to speed up NNLMs consists in converting them into standard count-based back-off models [25, 26].

The approach described in this work can be considered a simplification of [7]. It consists in using a sole NN that considers input contexts of different length in order to ease the use of the aforementioned fallback mechanism. In this way, when the normalization constant of the full word history is not found in the table, the model emulates a lower order one by replacing the farthest word of the past history for a 〈dummy〉 symbol. This procedure is applied, if required, until the word history is found in the table. This mechanism is guaranteed to succeed at least when descending down to bigrams, since an array of the same size as the lexicon suffices to cover all possible patterns.

The capability of the NN to emulate lower order models is obtained by properly training it with 〈dummy〉 inputs, as explained below. This technique reduces the number of model parameters and the computational resources, specially the training cost, initially required in [7] since, now, a single NN replaces several models.

Experiments have been performed for a pure LM task and for a machine translation system, where it is shown that the introduced approximation does not impact performance, while being faster to train and test than related models. Although some decisions have been made in these experiments (e.g. the shortlist approach), we have to remark that the proposed approach is not tied to these decisions and can be used in addition to other techniques.

## 2 Fallback Variable History NNLM

As stated in the introduction, our goal was to obtain a single NN able to estimate LMs of different orders to easily implement a fallback mechanism based on the memorization of precomputed normalization constants. This is achieved by using an input encoding able to represent contexts of variable size. The proposed model will be called “Fallback Variable History NNLM” (Fallback V-NNLM).

### 2.1 Emulating lower order *N*-grams by stochastic training

Skipping techniques, in the context of language modeling, consist in estimating several LMs based on different ways of removing words from the past history [27–32]. A natural way of applying this idea to NNLMs is by training a single NN but replacing random positions in the input representing the LM history by a new special null symbol 〈dummy〉. This idea can also be used to emulate lower order *N*-grams by skipping the required number of farthest words of the context, leading to a sole model, called Variable History NNLM (V-NNLM), that can emulate all the lower orders.

Thus, when requiring the evaluation of different word history lengths, a single NN suffices and a single NN needs to be trained instead of *N* − 1 NNs. To do so, patterns associated with several context sizes (containing the 〈dummy〉 symbol in zero or more positions of the input layer) should be used.

This can be easily achieved by stochastically perturbing the input. This is equivalent, but simpler, to replicate the training data for each *N*-gram order (replacing the farthest words by the 〈dummy〉 and shuffling the patterns afterwards). The *N*-gram order chosen for each training pattern has been stochastically sampled from an a priori distribution (a uniform distribution in the reported experiments).

Moreover, in order to achieve a better generalization, several models have been trained so that our final V-NNLM will be an ensemble of them. This ensemble could, in turn, be distilled into just one model afterwards using the ideas of [33].

### 2.2 The fallback strategy

Let us see how to combine V-NNLMs, based on emulating *N* − 1 NNLMs into one NNLM, and the idea of precomputing normalization constants. To this end, let us detail the fallback mechanism. It is convenient to distinguish the procedures required beforehand and at test time during decoding.

Once the V-NNLM has been trained, the softmax normalization constants of the most probable input contexts have to be precomputed and stored for each *N*-gram order down to bigrams. Bigrams deserve a special treatment since the entire set of contexts, which is of size |Ω|, is precomputed. In practice, we can take as the most probable contexts from the set of *N*-grams those with a count greater than a given threshold.

The required process to implement the fallback mechanism in test time is as follows:

1. Take into account the input of the V-NNLM consisting of an (*N* − 1)-gram.
2. Look for the constant associated with this input. If the constant is not found in the table associated with the input length, remove the last element from the input, and go back to 2.
3. Fill the input context with the 〈dummy〉 symbol in order to obtain the original input size and evaluate the V-NNLM up to the last hidden layer. Note that this step is compatible with the precomputation of values to speed up the first hidden layer as described, for instance, in [15].
4. Evaluate the neurons of the output layer for the set of desired words and use the constant found in step 2.

**Fig 1:**
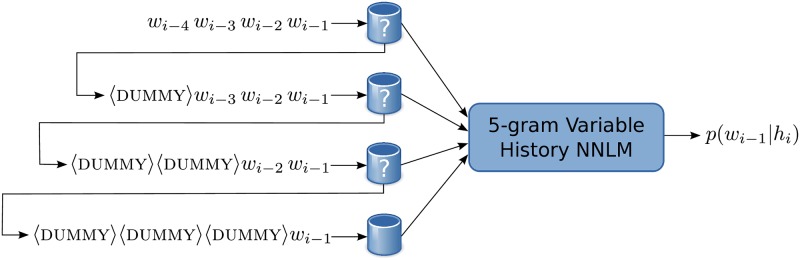
Scheme of a 5-gram Fallback Variable History NNLM (Fallback V-NNLM). It is composed of one 5-gram Variable History NNLM (V-NNLM) and four precomputed tables of constants. If the softmax normalization constant is found in the 5-gram table for the input *w*_*i*−4_*w*_*i*−3_*w*_*i*−2_*w*_*i*−1_, the query is processed and the probability *p*(*w*_*i*_∣*h*_*i*_) = *p*(*w*_*i*_∣*w*_*i*−4_*w*_*i*−3_*w*_*i*−2_*w*_*i*−1_) is computed. If not, the query is delegated to the same V-NNLM but with the input 〈dummy〉 *w*_*i*−3_ *w*_*i*−2_*w*_*i*−1_ at the 4-gram table, and so on.

Let us remind that point 2 is guaranteed to succeed since the table associated with bigrams contains the entire vocabulary. This whole process, illustrated in Fig 1, composes the Fallback Variable History NNLM (Fallback V-NNLM) model.

Compared with [7], a slight degradation in perplexity (PPL) and system performance could be expected from the fact that a 〈dummy〉 symbol is used to shorten the input context of the NN instead of training a dedicated model for each *N*-gram order. However, as observed in the experimental results, the performance loss is negligible in our case study and this is compensated by a simpler implementation, a reduced number of model parameters and a faster training process.

## 3 Experimental Setup I: Emulating lower order *N*-gram NNLMs

The following experiments will test the capability of V-NNLMs to emulate lower order *N*-grams.

### 3.1 Corpus

The proposed experimentation is based on the English part of the News-Commentary 2010 corpus, and the test sets of 2008 and 2010 editions of the *Workshop of Machine Translation* [34, 35]. Table 1 shows some statistics of this database (English part) which will be used to train the proposed LMs.

**Table 1:** Lines and words of the News-Commentary corpus (English part).

Set	# lines	# words
Training (News-Commentary 2010)	125.8K	2.9M
Validation (News 2008)	2.0K	49.7K
Test (News 2010)	2.5K	61.9K
Total	130.3K	3.0M

### 3.2 Model training

Many techniques aiming at reducing the computational cost of the output normalization layer are independent and compatible with the approach described in this work. We have combined one of them, namely, the shortlist approach [2, 11]. The shortlist approach consists in training the NNLM over a restricted vocabulary (known as *shortlist*) Ω′ ⊂ Ω composed by the most frequent words in the training corpora.

The vocabulary of the training set consists of |Ω| = 38 793 words. In our work, the shortlist is fixed to the |Ω′| = 20 000 most frequent words. In addition to the shortlist approach described previously for the output layer, we have measured the effect of using the same shortlist as the input vocabulary: every Out-Of-Shortlist (OOS) word (i.e. those from Ω − Ω′) is replaced by the OOS identifier at the input as well. We obtained indistinguishable results in previous experiments by using the whole vocabulary or the shortlist as the input vocabulary [36]. For that reason, we decided to use the restricted vocabulary (shortlist words plus a neuron associated with the OOS words) both at the input and at the output layer of the neural network.

OOS word probabilities can be computed by adding a new output neuron to compute *p*(*OOS*|*w*_*i*−*n*+1_ …*w*_*i*−1_). That is, *o*_*OOS*_ is the activation of the output neuron corresponding to all OOS words, and is an estimate of ∑_*w*∈*OOS*_ *p*(*w*|*w*_*i*−*n*+1_ …*w*_*i*−1_). This probability mass is then distributed among all OOS words. We have followed the approach described in [37] where a standard count-based unigram computed over OOS words is used in this distribution.

Backpropagation algorithm and L2 regularized cross-entropy loss function are used to train the networks: we have trained not only the V-NNLM but also a different NNLM for each *N*-gram order, from bigrams up to 5-grams, in order to evaluate the V-NNLM capability to emulate lower order models. Three different NNs have been trained for each model in order to perform an ensemble by means of a linear combination, leading to a total of 15 NNs. The three models combined in each case differ in the projection layer which comprise 128, 160, and 208 neurons, respectively. All of them have a hidden layer with 200 neurons. These numbers are based on previous experimentation [38], and were selected to improve PPL on the validation set.

In order to train the V-NNLM to deal with inputs of several context lengths, the context length of each training pattern is stochastically sampled from a uniform distribution ranging from 1 up to *N* − 1 and the appropiate number of 〈dummy〉 symbols are used to fill the input at the farthest positions.

After training the networks, the tables of precomputed softmax constants, for each possible *N*-gram order (ranging from bigram up to 5-grams) are precomputed. The size of these tables, which depend on the number of distinct *N*-grams found in the training data, are 20*K* for bigrams, 650*K* for trigrams, 1.79*M* for 4-grams and 2.42*M* for 5-grams, which are compactly stored by means of a trie data structure based on hashing.

Finally, in order to study the effect of the initialization weights of the different NNs, we have replicated the whole experiment 15 times by using, at each replica, a different set of random initialization seeds. Since each replica comprises the training of 15 NNs, the total number of trained NNs amounts to a total of 225.

### 3.3 Experiment: Emulating lower order *N*-grams with V-NNLMs

In order to evaluate the capability of V-NNLMs to emulate a set of standard NNLMs, we have measured the PPL of the validation and test sets by using the regular NNLMs of different *N*-gram orders, on the one hand, as well as the trained 5-gram V-NNLM, on the other. These values are shown in Table 2. Reported values correspond to the averaged PPL over 15 different experiments (as mentioned in the previous section), accompanied by the corresponding 95% confidence intervals. Let us remark that the V-NNLMs results are computed without using the fallback method.

**Table 2:** Averaged PPL measures for the News-Commentary validation and test sets. Measures given by regular NNLMs and by 5-gram V-NNLMs without using the fallback method. No combination with count-based models has been performed.

	Validation set PPL			
Model	*N*-gram order			
	2	3	4	5
Bigram NNLM	412.6 ± 1.0	–	–	–
Trigram NNLM	–	345.9 ± 0.9	–	–
4-gram NNLM	–	–	327.0 ± 1.1	–
5-gram NNLM	–	–	–	319.4 ± 1.0
V-NNLM	423.3 ± 1.3	356.3 ± 0.9	334.9 ± 0.8	326.5 ± 0.7
	Test set PPL			
Model	*N*-gram order			
	2	3	4	5
Bigram NNLM	407.7 ± 1.1	–	–	–
Trigram NNLM	–	338.6 ± 1.0	–	–
4-gram NNLM	–	–	320.6 ± 1.1	–
5-gram NNLM	–	–	–	312.6 ± 0.9
V-NNLM	418.2 ± 1.3	350.0 ± 1.0	329.4 ± 0.9	320.2 ± 0.8

Although the (averaged) PPL values for each *N*-gram NNLM are slightly better than the ones obtained by the corresponding V-NNLMs, as expected, the difference is not significant enough to harm the translation system performance, as we will observe in Section 4. In contrast, the advantages of using the V-NNLM include the fact of training and using a sole NN, instead of *N* − 1 NNs, to implement the fallback approach. We believe that these advantages clearly outweight the slightly observed degradation in PPL. We can also observe, from the confidence intervals, that the different initialization of the NN weights does not produce relevant differences in the observed PPL.

### 3.4 Experiment: Comparing standard LMs and Fallback V-NNLM

In this section, we are going to compare the PPL for the validation and test sets given by standard LMs and by the Fallback V-NNLM. The Fallback V-NNLM is composed by just one V-NNLM of maximum *N*-gram order and by *N* − 1 tables of precomputed softmax normalization constants.

For this experimentation, a count-based 4-gram has been trained with the SRI toolkit [39], and linearly combined with the Fallback V-NNLM as it is usually done with standard NNLMs. SRI has been configured to use modified Kneser-Ney smoothing with interpolation and unknown word probability computation. The linear combination weights are optimized to minimize the PPL in the validation set.

For comparison purposes, SRI models and a regular NNLM (where the normalization constant is actually computed) for each *N*-gram were also tested. The PPL measures, averaged over the 15 different experiments, are shown in Table 3. These values exhibit the excellent behaviour of the proposed model with respect to the corresponding SRI models in isolation. We can also observe that the gap from regular NNLMs to Fallback V-NNLMs is very small. Again, the low value of the confidence interval widths confirms the fact that the differences in the initialization of the NN weights does not produce noticeable differences in PPL. For that reason, the machine translation experiments of the following section will be conducted using only one of the 15 identical experimentation replicas.

**Table 3:** Averaged PPL measures for the News-Commentary validation and test sets. Measures given by SRI *N*-gram models, by regular NNLMs, and by the proposed Fallback V-NNLMs. Let us remark that both NNLMs and Fallback V-NNLMs are linearly combined with the 4-gram count-based model.

	Validation set PPL		
*N*-gram order	SRI	NNLM	F.V-NNLM
2	332	252.0 ± 0.2	252.6 ± 0.1
3	308	231.1 ± 0.3	237.9 ± 0.2
4	305	221.0 ± 0.4	233.0 ± 0.2
5	305	216.6 ± 0.4	232.2 ± 0.2
	Test set PPL		
*N*-gram order	SRI	NNLM	F.V-NNLM
2	409	244.8 ± 0.2	245.4 ± 0.1
3	377	223.4 ± 0.3	230.3 ± 0.2
4	297	213.8 ± 0.4	225.8 ± 0.2
5	297	209.0 ± 0.3	225.0 ± 0.3

## 4 Experimental Setup II: Models in a machine translation system

In this experimental framework, the proposed Fallback V-NNLM is used for a translation task.

### 4.1 Corpus

The experiments were also performed with the Spanish-English task of the News-Commentary 2010 corpus, from the *Workshop of Machine Translation 2010* (WMT’10) [35]. Statistics from this corpus are shown in Tables 1 and 4. These numbers were computed after cleaning, tokenization and lowercase preprocessing. The tokenization step was carried out by using the script tokenizer.perl from the WMT’10. The English vocabulary was extracted from the 80.9K sentences with lengths up to 40 words. The News2008 set was used as a development set; the News2009 set was used as an internal test set, for comparison purposes between systems. Finally, the News2010 set was used as a final test to measure the generalization ability of the full experimentation.

**Table 4:** Statistics of the bilingual Spanish-English task of the News-Commentary 2010 corpus.

Corpora	Spanish		English	
	# lines	# words	# lines	# words
News2008	2.0K	52.6K	2.0K	49.7K
News2009	2.5K	68.0K	2.5K	65.6K
News2010	2.5K	65.5K	2.5K	61.9K

### 4.2 Baseline translation system

The baseline translation experiments follow the well established phrase-based statistical machine translation approach [40] where the atomic units to be translated are whole sequences of words. It has been trained by using the open-source machine translation toolkit Moses [41] in its standard setup and using the configuration by default.

The word alignments to extract phrases have been obtained using Giza++ [42] with the heuristic grow-diag-final-and.

Besides the phrase table, phrase-based translation requires a language model of the target language. To this end, statistical *N*-gram LMs were trained with the SRI toolkit [39].

The overall trained system is composed by 14 models which are combined following the maximum entropy approach:

- Seven reordering models corresponding to the msd-bidirectional-fe.
- Language model of the target language.
- Four translation models: inverse phrase translation probability, inverse lexical weighting, direct phrase translation probability, direct lexical weighting.
- Number of phrases penalty.
- Number of words penalty.

All systems were optimized using the MERT procedure [43] on the News2008 set. A detailed description of the translation system can be found in [44].

Table 5 shows the obtained baseline performance for the News2010 test set, along with the average time to decode each sentence, using Moses and the April-ANN toolkit (https://github.com/april-org/april-ann). All the numbers are computed over lower-cased and tokenized sentences.

**Table 5:** Baseline results using Moses and our decoder for the News2010 test set. Results using a count-based 4-gram as the target language model. Time is the average decoding time (in seconds) per sentence measured on an Intel(R) Core(TM) i5 CPU 750 @2.67GHz CPU with 16GB RAM.

System	BLEU	TER	Time (s/sentence)
Moses	22.6	57.8	0.6
Our system	22.7	57.8	0.4

### 4.3 Translation experiments

The LMs of the target language were trained, for the baseline system, with the SRI toolkit [39]. In this section, the same NNLM and Fallback V-NNLM connectionist LMs described in Section 3, trained using the April-ANN toolkit, have been used to rerank a list of *n*-best hypotheses (in this experiment, *n* = 1000) generated by our system. Table 6 shows the BLEU and TER of the translations given by the systems, after reranking, along with the decoding times, computed as the average seconds per sentence of the translation plus the rescoring step times. All the numbers are computed over lower-cased and tokenized sentences.

**Table 6:** BLEU and TER for the News2010 test set for NNLM and Fallback V-NNLM models, for different *N*-gram orders. Time is computed as the average seconds per sentence of the translation plus the rescoring step times.

	BLEU		TER		Time (sec./sentence)	
*N*-gram order	NNLM	F.V-NNLM	NNLM	F.V-NNLM	NNLM	F.V-NNLM
2	22.9	22.9	57.5	57.5	0.5	0.5
3	23.3	23.1	57.3	57.4	0.7	0.5
4	23.4	23.2	57.2	57.4	1.5	0.6
5	23.2	23.1	57.3	57.4	2.6	0.6

In the case of NNLMs, we have taken advantage of the tables of normalization constants precomputed for the Fallback V-NNLM model. Note that this is a gain over conventional NNLM implementations relating the computational cost. Therefore, when the softmax constant is not found, it is computed exactly and memorized for future use during the decoding procedure for the same sentence, while the Fallback V-NNLM applies the fallback mechanism in that case.

The performance shows negligible differences between the Fallback V-NNLMs and NNLMs in both BLEU and TER. Regarding decoding times, observe that the use of precomputed values for the regular NNLMs leads to identical computations as the Fallback V-NNLMs since the entire vocabulary is precomputed. Otherwise stated, they are equivalent models for bigrams and lead to the very same results. When using trigrams, Fallback V-NNLMs are slightly faster and this difference in time increases as the *N*-gram order grows.

## 5 Conclusions and future work

This work presents a connectionist language model, called Fallback V-NNLM, that addresses the computational cost, during the evaluation phase, caused by the softmax activation output. Although many techniques have been proposed in the literature to tackle this problem, it cannot be considered a solved issue and, indeed, none of the proposed techniques has been adopted as standard by the community. The approach proposed in this work does not pretend to solve this problem in its generality but, rather, to provide an effective way to address it in some particular, albeit quite relevant, cases.

This model is composed of a unique NN, called V-NNLM, which can emulate lower order NNLMs by varying the context input. This model is used along with a collection of tables of precomputed softmax normalization constants in order to avoid the computation of the entire set of output neurons during decoding. When a normalization constant is not found, a fallback mechanism is followed. The capability of V-NNLMs to emulate lower order NNLMs greatly simplifies the use of this fallback mechanism.

As other techniques based on precomputing some values ahead of time, our approach has scalability problems with the *N*-gram order and with the lexicon size. However, compared with the conversion into standard count-based back-off models [25, 26], we can mention that a single precomputed constant in our approach is able to deal with a number of transitions equal to the lexicon size.

Experimental evidence on a statistical machine translation task conducted on the bilingual Spanish-English part of the News corpus shows a considerable speed-up when using NNLMs at the expense of a negligible loss in performance.

We believe that the proposed Fallback V-NNLM model makes the integration of NNLMs into the decoding stage (instead of rescoring *n*-best lists) much more feasible.

The proposed technique is compatible with many other techniques used both in training and evaluation, such as Noise-Contrastive Estimation [10], the shortlist approach [2, 11] (already used in the experiments), or the use of deep architectures [45], to mention a few.
